# Evaluation of a nutrient-rich food index score in the Netherlands

**DOI:** 10.1017/jns.2015.4

**Published:** 2015-04-20

**Authors:** Diewertje Sluik, Martinette T. Streppel, Linde van Lee, Anouk Geelen, Edith J. M. Feskens

**Affiliations:** Division of Human Nutrition, Wageningen University, PO Box 8129, 6700 EV Wageningen, the Netherlands

**Keywords:** Nutrient density, Nutrient profiling, Nutrient-rich food scores, Energy density, Dutch Healthy Diet Index, Diet quality, DHD-index, Dutch Healthy Diet Index, DNFCS, Dutch National Food Consumption Survey, DV, daily value, HEI-2005, Healthy Eating Index 2005, LIM, limited nutrient score, NR, nutrient-rich score, NRF, nutrient-rich food, RDA, recommended daily allowance, STB, standardised regression coefficient

## Abstract

Nutrient-rich food (NRF) index scores are dietary quality indices based on nutrient density. We studied the design aspects involved in the development and validation of NRF index scores, using the Dutch consumption data and guidelines as an example. We evaluated fifteen NRF index scores against the Dutch Healthy Diet Index (DHD-index), a measure of adherence to the Dutch dietary guidelines, and against energy density. The study population included 2106 adults from the Dutch National Food Consumption Survey 2007–2010. The index scores were composed of beneficial nutrients (protein, fibre, fatty acids, vitamins, minerals), nutrients to limit (saturated fat, sugar, Na) or a combination. Moreover, the influence of methodological decisions was studied, such as the choice of calculation basis (100 g or 100 kcal (418 kJ)). No large differences existed in the prediction of the DHD-index by the fifteen NRF index scores. The score that best predicted the DHD-index included nine beneficial nutrients and three nutrients to limit on a 100-kcal basis, the NRF9.3 with a model *R*^2^ of 0·34. The scores were quite robust with respect to sex, BMI and differences in calculation methods. The NRF index scores were correlated with energy density, but nutrient density better predicted the DHD-index than energy density. Consumption of vegetables, cereals and cereal products, and dairy products contributed most to the individual NRF9.3 scores. In conclusion, many methodological considerations underlie the development and evaluation of nutrient density models. These decisions may depend upon the purpose of the model, but should always be based upon scientific, objective and transparent criteria.

Nutrient profiling is defined as the science of categorising foods according to their nutritional composition^(^^1^^)^. Nutrient profile models enable consumers to identify foods that provide optimal nutrition at an affordable cost^(^^2^^)^. The main aim of nutrient profiling is to stimulate health and prevent diseases and its use has many possible applications, including food-based dietary guidelines, food labelling and health claims.

Nutrient-rich food (NRF) index scores are one method of nutrient profiling and are based on nutrient density. Nutrient density is the ratio of the nutrient composition of a food to the nutrient requirements of the human consumer^(^^3^^)^. The development of NRF index scores involves several methodological issues, including the selection of key nutrients, the choice of recommended daily allowances (RDA), and the basis of calculation (per 100 g, 100 kcal (418 kJ), or portion sizes). It is critical that the development and the validity of the scores are tested based on objective and transparent criteria. Fulgoni *et al.*^(^^1^^)^ have developed a number of index scores and validated them against the Healthy Eating Index 2005 (HEI-2005) in the US population. The NRF9.3 index, based on nine nutrients to encourage and three nutrients to limit, explained the highest percentage of variation from the HEI-2005.

In order to provide an evidence-based model, it is important that nutrient profile models are tested for their reliability and validity. We have previously evaluated the NRF index in terms of association with all-cause mortality and CVD among 4969 individuals aged 55 years and older from the Rotterdam Study. The NRF9.3 index showed to be inversely related to overall mortality, but not significantly with incident CVD^(^^4^^)^. To test the performance of nutrient profile models such as the NRF index, they should also be compared with each other and with other measures of a healthy diet. Moreover, nutrient profile models should ideally provide additional information next to the energy density^(^^5^^)^.

The present study will evaluate the design aspects involved in the development and validation of nutrient profile models, using the Dutch food consumption data and guidelines as an example. Within the Dutch National Food Consumption Survey (DNFCS) 2007–2010, we will test several NRF index scores, based on European RDA, and using the Dutch Healthy Diet Index (DHD-index), a measure of adherence to the Dutch guidelines for a healthy diet^(^^6^^)^. Furthermore, the association between the NRF index scores and energy density will be evaluated.

## Experimental methods

### Study design and population

The NRF index scores were evaluated within the DNFCS 2007–2010^(^^7^^)^. The DNFCS was conducted among children and adults aged 7–69 years in the Netherlands, excluding pregnant and lactating women and institutionalised individuals. The survey population was representative of the Dutch population with regard to age and sex within each age group, region, degree of urbanisation and educational level. In total, 5502 individuals aged 7–69 were invited, of which 3819 agreed to participate. For the present study, all subjects of 19 years and older (1055 men and 1051 women) were included. The present study was conducted according to the guidelines laid down in the Declaration of Helsinki. Written informed consent was obtained from all subjects.

### Dietary and covariate assessment

Data were collected by means of a general questionnaire and through two non-consecutive 24-h dietary recalls from March 2007 to April 2010. For the latter, each individual was interviewed twice with an interval of 2–6 weeks. The recalls were spread equally over all days of the week and seasons. The two 24-h dietary recalls were conducted by telephone using the computer-directed interview program EPIC-SOFT^(^^8^^,^^9^^)^. Consumption data were linked to the 2011 Dutch food composition database^(^^10^^)^ and averaged over 2 d. This food composition database included all nutrients tested. In addition, we estimated added sugar intake using criteria from the International Choices Programme, the Danish food composition table, and information from labels and recipes^(^^11^^,^^12^^)^. Added sugar was defined as all mono- and disaccharides added during food manufacturing and preparation. Raw, white and brown sugar, honey and syrups are assumed to be added during food preparation and thus considered as added sugars. Added sugars do not include naturally occurring mono- and disaccharides found in unprocessed products, fruit concentrates and bread, and lactose in dairy products. Foods were organised into sixteen food groups by the EPIC-SOFT classification. Weight and height were self-reported. The general questionnaire assessed age, sex, educational attainment, occupational status, smoking status and physical activity.

### Nutrient-rich food index scores evaluated

Table 1 gives an overview of the tested NRF index scores. These scores were based upon several nutrient profile models previously investigated by Drewnowski *et al.*^(^^13^^)^. The positive scores included protein, dietary fibre and a range of micronutrients; the negative scores comprised saturated fat, Na and total sugar as nutrients to limit.

**Table 1.** Tested nutrient-rich foods scores

Score	Macronutrients	Vitamins	Minerals	Nutrients to limit
NR6	Protein, dietary fibre	A, C	Ca, Fe	
NR9	Protein, dietary fibre	A, C, E	Ca, Fe, Mg, K	
NR11	Protein, dietary fibre	A, C, E, B_12_	Ca, Fe, Mg, K, Zn	
NR15	Protein, dietary fibre, monounsaturated fat	A, C, D, E, B_1_, B_2_, B_12_, folate	Ca, Fe, K, Zn	
NR18	Protein, dietary fibre, monounsaturated fat, linoleic acid, α-linolenic acid, fish fatty acids (EPA + DHA)	A, C, D, E, B_1_, B_2_, B_12_, folate	Ca, Fe, K, Zn	
NR19	Protein, dietary fibre, monounsaturated fat, linoleic acid, α-linolenic acid, fish fatty acids (EPA + DHA)	A, C, D, E, B_1_, B_2_, B_12_, folate	Ca, Fe, Mg, K, Zn	
NR20	Protein, dietary fibre, monounsaturated fat, linoleic acid, α-linolenic acid, fish fatty acids (EPA + DHA)	A, C, D, E, B_1_, B_2_, B_6_, B_12_, folate	Ca, Fe, Mg, K, Zn	
LIM3				Saturated fat, total sugar, Na
NRF6.3	Protein, dietary fibre	A, C	Ca, Fe	Saturated fat, total sugar, Na
NRF9.3	Protein, dietary fibre	A, C, E	Ca, Fe, Mg, K	Saturated fat, total sugar, Na
NRF11.3	Protein, dietary fibre	A, C, E, B_12_	Ca, Fe, Mg, K, Zn	Saturated fat, total sugar, Na
NRF15.3	Protein, dietary fibre, monounsaturated fat	A, C, D, E, B_1_, B_2_, B_12_, folate	Ca, Fe, K, Zn	Saturated fat, total sugar, Na
NRF18.3	Protein, dietary fibre, monounsaturated fat, linoleic acid, α-linolenic acid, fish fatty acids (EPA + DHA)	A, C, D, E, B_1_, B_2_, B_12_, folate	Ca, Fe, K, Zn	Saturated fat, total sugar, Na
NRF19.3	Protein, dietary fibre, monounsaturated fat, linoleic acid, α-linolenic acid, fish fatty acids (EPA + DHA)	A, C, D, E, B_1_, B_2_, B_12_, folate	Ca, Fe, Mg, K, Zn	Saturated fat, total sugar, Na
NRF20.3	Protein, dietary fibre, monounsaturated fat, linoleic acid, α-linolenic acid, fish fatty acids (EPA + DHA)	A, C, D, E, B_1_, B_2_, B_6_, B_12_, folate	Ca, Fe, Mg, K, Zn	Saturated fat, total sugar, Na

NR, nutrient-rich score; LIM, limited nutrient score; NRF, nutrient-rich foods score.

RDA as set by the European Union^(^^14^^)^ as well as the labelling reference intake values as set by the European Food Safety Authority were used as reference daily values (DV)^(^^15^^–^^18^^)^ (Table 2). The percentage of reference DV for each nutrient was capped at 100% DV to avoid overvaluing food items that provide very large amounts of a single nutrient, such as fortified foods^(^^3^^)^.

**Table 2.** Recommended daily values (RDV) and maximum daily values (MDV) based on an intake of 2000 kcal (8370 kJ) per d for selected nutrients by the European Food and Safety Authority (EFSA)

Nutrient	RDV	MDV
Nutrient-rich components		
Protein (g)	57*	
Dietary fibre (g)	25†	
Monounsaturated fat (g)	20‡	
Linoleic acid (g)	10‡	
α-Linolenic acid (g)	2‡	
Fish fatty acids (EPA + DHA) (mg)	250‡	
Vitamin A (RE)	800§	
Vitamin C (mg)	80§	
Vitamin D (μg)	5§	
Vitamin E (mg)	12§	
Vitamin B_1_ (mg)	1·1§	
Vitamin B_2_ (mg)	1·4§	
Vitamin B_6_ (mg)	1·4§	
Vitamin B_12_ (μg)	2·5§	
Folate (μg)	200§	
Ca (mg)	800§	
Mg (mg)	375§	
Fe (mg)	14§	
K (mg)	2000§	
Zn (mg)	10§	
Nutrients to limit		
Saturated fat (g)		20‡‖
Sugar (g)		
Total		90‖
Added		45‖
Na (mg)		2400‖

RE, retinol equivalents.

* EFSA Panel on Dietetic Products, Nutrition and Allergies^(^^15^^)^.

† EFSA Panel on Dietetic Products, Nutrition and Allergies^(^^17^^)^.

‡ EFSA Panel on Dietetic Products, Nutrition and Allergies^(^^18^^)^.

§ European Commission^(^^31^^)^.

‖ EFSA Panel on Dietetic Products, Nutrition and Allergies^(^^16^^)^.

### Calculation of the nutrient-rich food index scores

First, the scores were calculated for each food item per 100 kcal^(^^10^^)^. Subsequently, these food scores were converted into individual scores by multiplying the amount of energy consumed of each item, in 100-kcal units, by the nutrients to encourage (nutrient-rich; NR) scores and then summing these scores for each subject. Next, the NR index scores were divided by the number of 100-kcal units of the subjects’ total energy intake to provide a ‘weighted average’ score. For the nutrients to limit (LIM) score, the same approach was used.

The algorithms used to calculate the NRF index scores evaluated are listed in Table 3 and are based on sums of nutrients where all nutrients were equally weighted^(^^1^^)^. The algorithms which combined positive nutrients and nutrients to limit were based on subtracting the negative from the positive subscore, rather than a ratio between the two^(^^1^^)^. Moreover, the scores were calculated per 100 kcal, since this led to the highest percentage of variance accounted for in previous validation studies^(^^19^^)^.

**Table 3.** Overview of nutrient-rich foods algorithms^(^^3^^)^

Model	Algorithm	Comment
NR*n*_100 kcal_	∑_I_ = 1 – *n* (Nutrient_i_/RDV_i_) × 100	Nutrient_i_ = content of nutrient i in 100-kcal (418-kJ) edible portion; RDV_i_ = recommended daily values for nutrient i
LIM3_100 kcal_	∑_i_ = 1–3 (Nutrient_i_/MDV_i_) × 100	Nutrient_i_ = content of limiting nutrient i in 100-kcal (418-kJ) edible portion; MDV_i_ = maximum daily values for nutrient i
NRF*n*.3_100 kcal_	NR*n *– LIM3	Difference between sums

NR*n*, nutrient-rich score consisting of *n* beneficial nutrients, dependent on the tested NRF score; LIM3, limited nutrient score, consisting of three nutrients to limit; NRF, nutrient-rich foods score.

### Dutch Healthy Diet Index

The NRF index scores were evaluated against the DHD-index. The DHD-index is an *a priori* dietary index developed by van Lee *et al.*^(^^6^^)^ to measure adherence to the Dutch Guidelines for a Healthy Diet as proposed by the Health Council of the Netherlands in 2006^(^^20^^)^ (Table 4). These guidelines include recommendations on physical activity, and consumption of vegetables, fruit, dietary fibre, fish, SFA, *trans*-fatty acids, Na, alcohol, and the number of consumption occasions with foods and beverages that contain easily fermentable sugars and drinks that are high in food acids. The DHD-index is a continuous score with ten components based on adherence to the aforementioned guidelines, for all components a maximum of 10 points can be allotted. For these analyses, the physical activity component was not included, resulting in a maximum score of 90.

**Table 4.** Components of the Dutch Healthy Diet Index and their cut-off (maximum score) and threshold (minimum score) values^(^^6^^)^

Components	Minimum score (=0)	Maximum score (=10)
1. Physical activity (week)	0 activities*	≥5 activities
2. Vegetables (d)	0 g	≥200 g
3. Fruit + fruit juices (d)†	0 g	≥200 g
4. Fibre (d)	0 g/4·2 MJ	≥14 g/4·2 MJ
5. Fish (d)‡	0 mg EPA + DHA	≥450 mg EPA + DHA
6. SFA (d)	≥15% energy	<10% energy
7. *Trans*-fatty acids (d)	≥1% energy	<1% energy
8. Consumption occasions (d)§	>7 occasions	≤7 occasions
9. Na (d)	≥2·52 g	<1·68 g
10. Alcohol (d)	Men: ≥6 drinks Women: ≥4 drinks	Men: ≤2 drinks Women: ≤1 drink

* Activities were at least moderately intensive and minimally 30 min.

† Maximum of 100 g of juice (six specific types) could be included.

‡ Fish intake was estimated based on dietary fish fatty acids (EPA + DHA) and fish oil capsules.

§ The number of consumption occasions was defined as the number of hours where at least one food drink with a pH < 5·5 and total acidity >0·5% was consumed.

### Statistical analyses

All statistical analyses were performed with SAS software (version 9.2; SAS Institute, Inc.). Spearman correlation coefficients between all NRF index scores and the DHD-index were calculated. Separate regression analyses were conducted using the DHD-index as the dependent variable and the NRF index scores as independent variable, testing one score at a time. Proportion of explained variance (score *R*^2^ and model *R*^2^) and standardised regression coefficients (STB) were estimated. Models were adjusted for age and sex.

Several methodological issues in the development and validation of NRF index scores were considered. For that reason, multiple sensitivity analyses were performed to assess the robustness of the results. The regression analyses were conducted separately for men and women, for normal-weight (BMI < 25 kg/m^2^) and overweight subjects (BMI > 25 kg/m^2^), with scores based on added sugar instead of total sugar, with uncapped scores, i.e. the reference DV was not capped at 100%, with scores based on calculations per 100 g instead of 100 kcal, with scores based on the American RDA, and finally, with scores based on means of the positive and negative nutrients instead of the sums of the nutrients.

Next, the relationship with energy density of the diet was explored by Spearman correlation coefficients and linear regression. Energy density was defined as kcal/g, using four definitions: (1) including all foods and beverages; (2) including all foods and energy-containing beverages (milk, juice, soft drinks and alcoholic beverages); (3) including all foods (excluding all beverages); and (4) including all foods and milk (excluding all other beverages)^(^^21^^)^.

The NRF index score with the highest proportion of explained variance was used to score all foods. Mean index scores on a food-group level as well as the mean contribution (%) of food groups to the individual weighted scores were calculated to give insight into the contribution of food groups to the scores.

The regression analyses were weighted for small deviances in sociodemographic characteristics, day of the week and season of data collection to yield results that are representative for the Dutch population, for all days of the week and all seasons. These weighting factors were derived from Dutch census data from 2008 as the reference population and were created in an iterative process^(^^22^^)^.

## Results

Correlations between the scores (excluding LIM) ranged from 0·76 to 0·99. No significant correlations were observed between the positive scores (NR6 to NR20) and the LIM. LIM was inversely correlated to the NRF scores, ranging from –0·05 with NRF18.3 to –0·27 with NRF6.3 (data not shown).

**Fig. 1.**
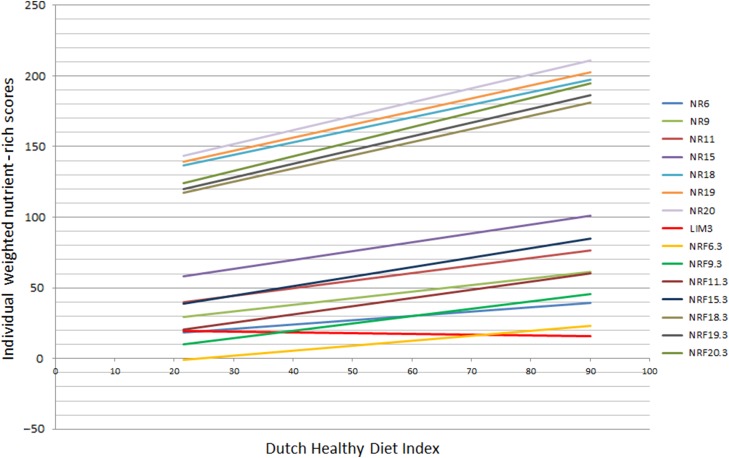
Crude linear associations between nutrient-rich foods index scores and the Dutch Healthy Diet Index in 2106 adults from the Dutch National Food Consumption Survey 2007–2010. NR, nutrient-rich score; LIM, limited nutrient score; NRF, nutrient-rich foods score.

Fig. 1 displays the crude linear associations between the NRF index scores and the DHD-index. Table 5 shows the results of the correlation coefficients and linear regression analyses of the tested scores on the DHD-index. No large differences in linear associations, correlations or prediction of the DHD-index existed between the fifteen tested NRF index scores. Of the positive NR scores, the NR9 showed the highest proportion of explained variance. The NRF scores combining positive nutrients and nutrients to limit were most predictive. The NRF9.3 showed best prediction with score *R*^2^ = 0·32 and a STB of 0·57. The correlation coefficient between NRF9.3 and the DHD-index was 0·60. NRF9.3 was most predictive of the DHD-index in both men and women, although the prediction was higher in women. STB was 0·52 with a score *R*^2^ of 0·27 in men, whereas in women, the STB was 0·62 with a score *R*^2^ of 0·38. Women had a higher mean NRF9.3 score with a smaller range compared with men: 23·4 (range –2·3 to 82·0) in women and 22·0 (range 0·15 to 190) in men. NRF9.3 was also most predictive of the DHD-index in both normal-weight and overweight and obese subjects; prediction was higher among overweight and obese subjects. In normal-weight subjects (BMI < 25 kg/m^2^; *n* 1010), STB was 0·52 (score *R*^2^ = 0·27) and in overweight and obese subjects (BMI ≥ 25 kg/m^2^; *n* 1095), STB was 0·61 (score *R*^2^ = 0·37). Adjustment for BMI did not alter the results.

**Table 5.** Mean nutrient-rich foods index scores based on sums per 100 kcal (418 kJ) by sex and linear regressions of nutrient-rich scores on the Dutch Healthy Diet Index (DHD-index) in 2106 adult men and women from the Dutch National Food Consumption Survey 2007–2010

	All (*n* 2106)		Men (*n* 1055)		Women (*n* 1051)		DHD-index	Linear regression on DHD-index*			
Scores	Mean	sd	Mean	sd	Mean	sd	Spearman's ρ	β	STB	*R*^2^ score	*R*^2^ model
NR6	29·0	8·5	27·8	8·1	30·5	8·6	0·50†	0·74	0·46	0·21	0·24
NR9	45·4	12·0	43·5	11·5	47·2	12·0	0·56†	0·60	0·52	0·25	0·27
NR11	58·2	15·6	56·2	15·1	60·2	15·6	0·48†	0·39	0·45	0·20	0·23
NR15	79·7	20·0	77·5	19·5	82·0	20·0	0·44†	0·28	0·40	0·16	0·20
NR18	167·0	27·9	163·1	27·4	170·9	27·5	0·42†	0·20	0·41	0·17	0·20
NR19	171·0	28·5	167·1	28·1	175·1	28·2	0·43†	0·20	0·42	0·18	0·21
NR20	177·2	29·8	173·2	29·2	181·3	29·6	0·44†	0·20	0·43	0·18	0·22
LIM3	22·6	4·5	21·5	4·3	23·8	4·3	−0·26†	−1·22	−0·26	0·07	0·11
NRF6.3	6·4	8·8	6·0	8·5	6·7	9·1	0·57†	0·84	0·54	0·29	0·31
NRF9.3	22·7	12·3	22·0	12·0	23·4	12·6	0·60†	0·63	0·57	0·32	0·34
NRF11.3	35·5	15·7	34·7	15·3	36·4	16·0	0·52†	0·43	0·49	0·24	0·27
NRF15.3	57·1	20·1	56·0	19·6	58·2	20·4	0·47†	0·30	0·44	0·19	0·22
NRF18.3	144·3	27·5	141·6	26·9	147·1	27·7	0·45†	0·22	0·44	0·19	0·22
NRF19.3	148·4	28·2	145·6	27·6	151·3	28·4	0·46†	0·22	0·45	0·20	0·23
NRF20.3	154·6	29·6	151·7	28·8	157·5	29·9	0·47†	0·21	0·46	0·21	0·24

STB, standardised regression coefficient; NR, nutrient-rich score; LIM, nutrients to limit; NRF, nutrient-rich foods score.

* Values are weighted for demographic factors, season and weekday, and adjusted for age and sex.

† *P* < 0·05.

Results were similar when using American RDA and scores based on means instead of sums: NRF9.3 showed best prediction, with a STB of comparable magnitude. The scores based on added sugar showed less prediction than scores based on total mono- and disaccharides; however, NRF9.3 still explained most variation: STB for NRF9.3 based on added sugar was 0·49 and score *R*^2^ = 0·24. Next, the regression analyses with uncapped scores showed that when linoleic acid, α-linoleic acid and fish fatty acids were included in the models, some nutrients contributed unduly to the score and scores were very high; NRF20.3 score was, for example, 4067 (sd 1838) in men and 3948 (sd 1849) in women. For all scores, the percentage explained variance of the DHD-index was lower using uncapped instead of capped scores. Best prediction in uncapped scores was seen for NRF9.3 with a STB of 0·50 and a score *R*^2^ of 0·25 adjusted for age and sex. Moreover, using 100 g as a calculation basis rather than 100 kcal, LIM3 showed the best prediction with a STB of –0·44 and a score *R*^2^ of 0·19.

Table 6 shows Spearman's correlation coefficients of the NRF scores and the DHD-index with energy density according to four definitions. Positive and combined NR scores correlated negatively with energy density; the LIM3 score positively. In contrast, energy density including all foods and all energy-containing beverages (definition 2) correlated positively with most scores. Highest correlations were found for NR9 and NR11 with energy density including food only (definition 3). Linear regression of energy density including all foods and milk (definition 4) showed a low prediction of the NR scores. The prediction of the DHD-index by energy density (definition 4) was even lower with a score *R*^2^ of 0·03.

**Table 6.** Spearman correlation coefficients (ρ) between nutrient-rich foods index scores based on sums per 100 kcal (418 kJ), the Dutch Healthy Diet Index (DHD-index) and energy density and linear regressions of nutrient-rich foods index scores, the DHD-index and energy density

	Energy density*	Energy density†	Energy density‡	Energy density§	Linear regression on energy density*§			
Scores	ρ	ρ	ρ	ρ	β	STB	*R*^2^ score	*R*^2^ model
NR6	−0·49‖	0·01	−0·64‖	−0·44‖	−0·04	−0·37	0·14	0·14
NR9	−0·51‖	0·03	−0·67‖	−0·46‖	−0·03	−0·39	0·15	0·15
NR11	−0·47‖	0·01	−0·67‖	−0·49‖	−0·03	−0·41	0·17	0·17
NR15	−0·42‖	0·03	−0·63‖	−0·48‖	−0·02	−0·39	0·15	0·16
NR18	−0·34‖	0·16‖	−0·50‖	−0·37‖	−0·01	−0·33	0·11	0·11
NR19	−0·36‖	0·15‖	−0·51‖	−0·38‖	−0·01	−0·34	0·11	0·12
NR20	−0·36‖	0·14‖	−0·52‖	−0·39‖	−0·01	−0·34	0·12	0·13
LIM3	0·10‖	0·01	0·02	0·00	−0·01	−0·04	0·00	0·02
NRF6.3	−0·52‖	−0·02	−0·62‖	−0·41‖	−0·04	−0·34	0·12	0·12
NRF9.3	−0·52‖	0·01	−0·64‖	−0·43‖	−0·03	−0·36	0·13	0·13
NRF11.3	−0·49‖	0·00	−0·66‖	−0·48‖	−0·02	−0·40	0·16	0·16
NRF15.3	−0·43‖	0·02‖	−0·62‖	−0·47‖	−0·02	−0·38	0·15	0·15
NRF18.3	−0·35‖	0·16‖	−0·50‖	−0·37‖	−0·01	−0·32	0·10	0·11
NRF19.3	−0·37‖	0·15‖	−0·51‖	−0·38‖	−0·01	−0·33	0·11	0·12
NRF20.3	−0·37‖	0·13‖	−0·52‖	−0·39‖	−0·01	−0·34	0·11	0·12
DHD-index	−0·43‖	0·09‖	−0·49‖	−0·22‖	−0·01	−0·18	0·03	0·05

STB, standardised regression coefficient; NR, nutrient-rich score; LIM, nutrients to limit; NRF, nutrient-rich foods score.

* Energy density (kcal/g) including all foods and beverages.

† Energy density (kcal/g) including all food and energy-containing beverages, excluding water, tea and coffee.

‡ Energy density (kcal/g) including food only, excluding all alcoholic and non-alcoholic beverages and milk (beverages).

§ Energy density (kcal/g) including food and milk (beverages), excluding all other alcoholic and non-alcoholic beverages.

‖ *P* < 0·05.

Food groups that had the highest NRF9.3 index score on food-item level were vegetables and non-alcoholic beverages, followed by legumes, potatoes, and the group of fruits, nuts and olives (Table 7). Non-alcoholic beverages scored very high because nutrient and energy density are highly influenced by its water content. Food items that had lowest NRF9.3 scores were sugar and confectionery, cake and biscuits, and condiments and sauces. In contrast to the NRF9.3 index scores on food-item level, the individual NRF9.3 scores take into account the choice of food items and the amount eaten. Among the DNFCS study population, vegetables, cereals and cereal products, and dairy products had the largest contribution to the individual NRF9.3 scores. However, inter-individual variation was quite high.

**Table 7.** Mean NR9, LIM3 and NRF9.3 index scores on food-item level based on the 2011 Dutch Food Composition Table and mean contribution (%) of food groups to the individual weighted scores in 2106 adults from the Dutch National Food Consumption Survey 2007–2010

				Percentage contribution of food groups to the individual weighted scores					
	Mean index scores on food-item level			NR9		LIM3		NRF9.3	
Food groups	NR9	LIM3	NRF9.3	Mean	sd	Mean	sd	Mean	sd
Potatoes and tubers	56·9	3·3	53·6	6	5	1	1	13	27
Vegetables	224·7	31·5	193·1	9	6	2	2	18	25
Legumes	77·9	19·7	58·3	0	2	0	1	1	4
Fruits, nuts and olives	72·8	21·6	51·2	7	7	4	5	10	14
Dairy products (milk, yogurt, cheese, desserts, cream)	63·3	47·5	15·8	19	10	24	1	15	28
Cereals (flour, pasta, rice, bread, cereals, dough)	28·7	21·2	7·4	16	7	14	6	18	27
Meat and meat products	52·6	52·8	−0·2	13	8	14	9	9	29
Total fish	69·4	44·0	25·4	2	4	1	3	2	6
Eggs and egg products	87·5	38·8	48·7	2	3	1	1	2	11
Fats (oil, butter, margarine, deep frying fat)	41·3	34·9	6·4	5	4	8	5	2	20
Sugar and confectionery (sugar, honey, jam, chocolate, confectionery, syrup, ice cream)	14·1	27·5	−13·4	3	4	8	7	−5	28
Cake and biscuits	20·0	30·2	−10·1	3	4	8	7	−2	10
Non-alcoholic beverages (juices, soft drinks, coffee, tea, water)	179·5	26·1	153·5	9	8	7	7	10	62
Alcoholic beverages (beer, wine, spirits, cocktails)	13·7	4·0	9·7	2	3	1	2	3	7
Condiments and sauces	47·5	73·2	−25·7	3	3	6	5	−1	10
Soups and bouillons	84·0	49·7	34·3	2	4	1	2	3	12
Miscellaneous (soya, dietetic products, snacks)	41·2	25·9	15·3	1	4	2	4	1	10

NR, nutrient-rich score; LIM, nutrients to limit; NRF, nutrient-rich foods score.

## Discussion

In this cross-sectional study, NRF index scores were related to adherence to the guidelines for a healthy diet as measured with the DHD-index in Dutch adults. No large differences existed in the prediction of the DHD-index by the fifteen tested NRF index scores. The NRF9.3 on a 100-kcal basis showed the highest prediction of the DHD-index with a model *R*^2^ of 0·34. The prediction of the NRF index scores was quite robust with respect to sex, BMI, using added or total mono- and disaccharides, using scores based on means instead of sums, and using US instead of European RDA. The NRF index scores were negatively correlated with energy density, but the prediction of the NRF index scores and the DHD-index by energy density was not high.

Of the fifteen tested scores, the prediction of the DHD-index was highest for the NRF9.3, with a *R*^2^ of 0·34. Fulgoni *et al.*^(^^1^^)^ have previously validated six NRF index scores against the HEI-2005 within the National Health and Nutrition Examination Survey 1999–2002. In that study, the NRF9.3 index based on 100 kcal best predicted the HEI-2005 with an *R*^2^ of 0·45. Compared with Fulgoni *et al.*^(^^1^^)^, the proportion of explained variance of the NRF index scores against the DHD-index was somewhat lower, but not to a great extent. This might be caused by the different study population or differences between the DHD-index and the HEI. In line with Fulgoni *et al.*^(^^1^^)^, we found no large variance in prediction between the tested NRF index scores. Furthermore, correlation coefficients between 0·5 and 0·7 seem typical for the reproducibility of nutrient intakes^(^^23^^)^. Thus, with a correlation coefficient of 0·60, the NRF9.3 index seemed to perform rather well.

The NRF index scores were inversely correlated to most definitions of energy density. Drewnowski *et al.*^(^^13^^)^ have previously shown that the relationship of nutrient profile models with energy density weakened as more nutrients were introduced into the model^(^^13^^)^. To provide additional information, the correlation between a nutrient profile model and energy density should ideally be low: high correlations with energy density indicate a high agreement between nutrient density and energy density^(^^5^^)^. When energy density was calculated including all food and energy-containing beverages (definition 2), correlations with nutrient density models were very low. Beverages are generally high in water and have low energy density compared with foods, although they contain energy. On the other hand, a nutrient density score by 100 kcal tends to assign the highest nutrient density values to beverages. Because non-alcoholic beverages are an important source of energy, this issue warrants attention in the further development of both energy and nutrient density models. The prediction of the DHD-index by energy density was much lower than by nutrient density, indicating that nutrient density models provide more information on diet quality than energy density models. However, ideally a prospective study linking nutrient density to health outcomes should be used to investigate whether nutrient density models provide additional information, independently from energy density.

Although the tested algorithms were highly correlated, it was shown that adding more vitamins and minerals to the model did not necessarily improve the prediction^(^^13^^)^. The choice of qualifying nutrients was based on limiting or shortfall nutrients in a population diet – including fibre, vitamins A, C and E, Ca, Mg and K – on one hand and additional nutrients of public health significance – including fibre, vitamin E and Mg – on the other hand^(^^24^^)^. This choice was mainly based upon the US population, whereas other nutrients might be of more importance in the Netherlands. Ca is not a shortfall nutrient due to the high dairy product consumption, whereas vitamins D and B_12_ and folate might classify more since food fortification with these nutrients is not allowed. Nevertheless, the prediction of the DHD-index did not differ to a great extent between the scores and NRF9.3 performed best in the Dutch population as well as in a US population. Which nutrients to include in a nutrient profile model also depends on the purpose of the model. A nutrient profile model for regulatory purposes might emphasise more the scientific basis and a model used for food labelling may include fewer nutrients in order to be efficient.

The NRF scores were summed and divided by 100-kcal units consumed to provide a weighted average score. Values based on portion sizes may provide a better way to communicate the concept of nutrient density to the consumer. However, the European Union lacks a harmonised standard definition of portion size, such as the reference amounts customarily consumed (RACC) servings in the USA^(^^13^^)^. Although models based on 100 g would seem to have universal appeal, model profiles based on 100 g of foods make no allowances for the fact that different foods and beverages are consumed in very different amounts^(^^3^^)^. Therefore, we based the NRF scores on 100 kcal. One caution is that scores based on 100 kcal have the effect of assigning the highest scores to foods with the highest water content and the lowest energy density, which may not necessarily indicate a higher diet quality. This is illustrated by the high scores for vegetables and non-alcoholic beverages in our study^(^^3^^)^.

The calculation of NRF scores also involves several issues. The prediction of the NRF score showed to be robust concerning the choice of RDA, using added or total sugar, and using means or sums of scores. The percentage of reference DV was capped at 100% DV to avoid overvaluing food items that provide very large amounts of a single nutrient, such as fortified foods^(^^3^^)^. The percentage explained variance of the DHD-index was lower using uncapped instead of capped scores. Moreover, the calculation of uncapped scores equals the calculation of nutrient density of the total diet, whereas the purpose of nutrient profiling is to be food-based.

The prediction of the DHD-index was higher in women than in men. The individual weighted nutrient density scores were also higher in women than in men, although the range was smaller. We have previously seen that the NRF9.3 was more strongly related to lower mortality risk in women than in men^(^^4^^)^. Using total sugar instead of added sugar in the calculation of the scores has been shown to be a reasonable option^(^^1^^)^. Indeed, in the present study the scores based upon total mono- and disaccharides performed slightly better than those using added sugars. The NRF scores were based upon recommended DV as set by the European Union^(^^14^^)^ as well as the labelling reference intake values as set by the European Food Safety Authority^(^^15^^–^^18^^)^. Some differences exist between these values and the dietary reference intakes published by the Institute of Medicine in the US DV for macronutrients are by and large the same, but the recommendations for most vitamins and minerals are slightly higher in the USA than in Europe. However, using American instead of European recommendations did not influence the prediction of the DHD-index.

Food items with the highest NRF9.3 index scores were vegetables, legumes, fruits, nuts and olives. When looking at the contribution of food groups to the individual NRF9.3 index scores in the study population, it was seen that vegetables, cereals and dairy products had the largest contribution. Within the Rotterdam Study, we have previously found that the NR9, LIM3 and NRF9.3 index scores were associated with all-cause mortality, but not with major CVD events^(^^4^^)^. We have discussed that this might have been caused by the fact that individual weighted NRF scores not only depend on the NRF score on the food-item level, but also on which products are eaten and in which amount. Thus, this aspect of the NRF index score warrants attention when testing and validating these scores.

The DHD-index is based upon the Dutch Guidelines for a Healthy Diet published by the Health Council of the Netherlands in 2006 and consists of ten components. In contrast to the HEI-2005, the DHD-index includes adherence to guidelines for physical activity, fish, *trans*-fatty acids, acidic foods and beverages, and alcohol. In the evaluation of the nutrient density scores, the physical activity component was omitted. The DHD-index includes two components for fruit and vegetable intake and does not include recommendations for grains, milk, meat and beans, and energy intake. Both the DHD-index and the HEI apply similar weights to all components. The HEI has been evaluated and it has been shown to be a valid measure of diet quality^(^^25^^,^^26^^)^. The DHD-index is the first and only instrument to measure adherence to the current Dutch dietary guidelines. The index showed to be a good measure of nutrient density of diets and was able to rank participants from the DNFCS according to their adherence to the guidelines^(^^6^^)^. Energy-adjusted intakes of folate, Fe, Mg, K, thiamin and vitamin B_6_ were positively associated with the DHD-index; unadjusted intakes of Ca and vitamin E showed an inverse association^(^^6^^)^. Next, the index was associated with the biomarkers serum carotenoids, EPA and DHA from phospholipids and urinary Na in the Dutch subsample of the European Food Consumption Validation Study^(^^27^^)^. Furthermore, higher scores for the DHD-index were associated with a lower mortality risk in the Rotterdam Study^(^^28^^)^, but not with a lower cancer risk in the Dutch subsample of the European Prospective Investigation into Cancer and Nutrition (EPIC-NL)^(^^29^^)^.

In conclusion, no large differences between fifteen NRF index scores and the prediction of the DHD-index were detected. The nutrient density models provided additional information on dietary quality compared with energy density. Many methodological issues underlie the development and evaluation of NRF index scores. At the moment, no standardised procedures for development, testing and validation exist^(^^30^^)^. These procedures may depend upon the purpose of the model, but it is vital that they are based on scientific, objective and transparent criteria in order to develop an evidence-based nutrient profile model.
